# Comparison of the efficiency of ultrasound-guided ESPB and TAPB on postoperative analgesia: a system review and meta-analysis

**DOI:** 10.3389/fmed.2025.1595778

**Published:** 2025-05-26

**Authors:** Lu Qian, Nian-qiang Hu, Qi-hong Shen, Kai Ni

**Affiliations:** ^1^Department of Anesthesiology, Sir Run Run Shaw Hospital, Affiliated with the Zhejiang University School of Medicine, Hangzhou, China; ^2^Department of Anesthesiology, Affiliated Hospital of Jiaxing University, Jiaxing, China; ^3^Department of Anesthesiology, Shulan (Hangzhou) Hospital, Shulan International Medical College, Zhejiang Shuren University, Hangzhou, China

**Keywords:** transversus abdominis plane block, erector spinae plane block, meta-analysis, ESPB, TAPB

## Abstract

**Background:**

This meta-analysis systematically evaluates the analgesic efficacy of two regional anesthesia techniques - transversus abdominis plane block (TAPB) and erector spinae plane block (ESPB) in abdominal surgical procedures.

**Methods:**

This PRISMA-compliant meta-analysis systematically queried PubMed, Embase, Web of science, and Cochrane library. Eligible studies were controlled clinical trials comparing ESPB and TAPB for postoperative analgesia, documenting pain scales, opioid use, and safety outcomes. Methodological rigor was evaluated per Cochrane criteria, with quantitative synthesis conducted via RevMan 5.4 using effect magnitudes (SMD/MD) and risk ratios (RR). Evidence certainty was graded using GRADE methodology.

**Result:**

Pooled data from 21 RCTs (*n* = 1,293 patients) revealed better pain control during the 24-h postoperative period in the ESPB groups (2-h: MD = −0.68, 95% CI [−1.04, −0.32], *p* < 0.05). Also, postoperative opioid consumption was significantly reduced in the ESPB group (MD = −1.25; 95% CI [−1.66 to −0.85]; *p* < 0.05). No significant differences were observed in complication occurrence (RR = 1.13, 95% CI [0.75, 1.71], *p* > 0.05).

**Conclusion:**

Current evidence indicates that ESPB demonstrates superior postoperative analgesic efficacy and reduced opioid requirements compared to TAPB, while maintaining comparable safety profiles.

**Systematic review registration:**

https://www.crd.york.ac.uk/PROSPERO/view/CRD42021275992.

## Introduction

Abdominal surgical procedures constitute a cornerstone of global surgical practice, with epidemiologic reports indicating a steadily escalating procedure volume accounting for 20–35% of all operative interventions annually (1). Contemporary surgical approaches, ranging from minimally invasive laparoscopy to conventional laparotomy, continue to confront substantial postoperative nociceptive burden. Previous studies documented 38–42% incidence of moderate-to-severe acute postsurgical pain (Visual Analog Scale ≥4) within 48 h post-procedure (2), a critical clinical determinant associated with functional recovery impairment (3) and elevated 30-day complication risks (4).

While neuraxial analgesia maintains its status as the reference standard for abdominal pain management (5), technical constraints (e.g., anticoagulation contraindications, anatomical complexity) limit its universal applicability. The advent of fascial plane blocks has revolutionized regional anesthesia paradigms since the seminal description of transversus abdominis plane block (TAPB) by Rafi in 2001 (6). This ultrasound-guided interfascial technique deposits local anesthetic between the transversus abdominis and internal oblique muscle layers, achieving somatic analgesia through blockade of thoracolumbar nerve branches (T6-L1) (7). Nevertheless, its inherent anatomical confinement precludes visceral nociception modulation-a critical limitation given that visceral afferents mediate 68% of post-laparotomy pain components. Erector spinae plane block (ESPB), first conceptualized in 2016 for chronic thoracic pain management (8), has emerged as a versatile truncal analgesia modality. Clinical series have validated its efficacy across diverse surgical contexts, from thoracic to pelvic procedures (9–11). Recent meta-analyses comparing ESPB and TAPB present conflicting conclusions: Matthew et al. demonstrated ESPB’s superiority in opioid-sparing effects (12), whereas Lin’s analysis found ESPB does not provide better clinical analgesia than the TAPB (13). Furthermore, the above meta-analyses had sample sizes.

Thus, the purpose of this review is to compare the efficacy of the ESPB with the TAPB in patients undergoing abdominal surgeries.

## Methods

### Study design and registration

Conducted per PRISMA 2020 guidelines, this pre-registered meta-analysis (PROSPERO CRD42021275992) adhered to systematic review standards.

### Information sources and search strategy

A systematic multistage search algorithm was executed across four electronic databases: PubMed, Embase, Cochrane library, and Web of science. The search chronology spanned from database inception to October 31, 2024, with no linguistic or publication status restrictions. The optimized Boolean syntax incorporated: MeSH terms: *“NerveBlock” [Mesh]*, *“Analgesia” [Mesh];* Free-text permutations: *(erector spinae OR ESP) AND (plane block OR fascial block)*, *(transversus abdominis OR TAP) AND (regional anesthesia OR nerve block)*; Procedure-specific filters: (*“abdominal surgery” [tiab] OR laparotom* [tiab] OR colectom*[tiab])*. An exemplar PubMed search strategy is detailed in Supplementary Data. Snowball searching was performed on included studies’ reference lists, supplemented by contact with corresponding authors for unpublished datasets.

### Study selection criteria

The inclusion criteria were formulated according to PICOS framework with the following operational definitions: Population (P): Patients ≥18 years undergoing elective procedures under general anesthesia; Intervention (I): Ultrasound-guided ESPB; Comparator (C): Ultrasound-guided TAPB; Outcomes (O): *Primary*: Pain score at 2-h postoperative; *Secondary*: Pain scores at 4 h, 6 h, 8 h, 12 h, and 24 h during postoperative period; intraoperative opioid consumption; incidence of procedure-related complications (vascular puncture, local anesthetic systemic toxicity), and postoperative nausea and vomiting (PONV); Study design (S): Parallel-group RCTs with ≥20 participants per arm. Exclusion criteria comprised: (1) Non-randomized designs (case series, editorials, narrative reviews); (2) Conference abstracts without peer-reviewed full texts; (3) Ongoing trials without primary outcome data; (4) Combined regional techniques (e.g., ESPB with paravertebral block).

### Data extraction protocol

Two researchers independently managed study selection: initial deduplication using EndNote; title/abstract screening for relevance; full-text review against inclusion criteria. Data extraction included: study characteristics (author, year, sample size); surgical/anesthesia details; complication rates (nerve block effects, and PONV). Discrepancies were resolved through consensus discussions.

### Risk of bias and evidence quality assessment

The Cochrane Review Manager (version 5.3) was employed to assess potential study biases. Two independent reviewers appraised trials based on:

- Selective outcome reporting
- Incomplete outcome data
- Evaluator/participant blinding status
- Allocation concealment methods
- Random sequence generation
- Other potential biases

The GRADE framework evaluated evidence certainty through six domains: study design, risk of bias, imprecision, inconsistency, indirectness, and other considerations. Evidence quality was stratified into four levels: very low, low, moderate, or high.

### Statistical analysis

Quantitative synthesis was performed using Review Manager 5.3. For dichotomous variables, pooled effects were expressed as risk ratios (RR) with 95% CIs. Continuous outcomes were analyzed through standardized mean differences (SMDs) or weighted mean differences (MDs), accompanied by 95% CIs. When studies reported continuous variables as medians with interquartile or min-max ranges, these values were converted to parametric measures using established transformation algorithms (14, 15).

The predefined statistical significance threshold was set at *α* = 0.05. Between-study heterogeneity was quantified using *I*^2^ statistics, with values exceeding 50% denoting substantial heterogeneity. Given the multiple sources of clinical heterogeneity arising from variations in surgical protocols and analgesic regimens, a random-effects model was uniformly implemented for pooled analyses irrespective of *I*^2^ statistic values. To explore potential sources of heterogeneity in primary outcome, we performed meta-regression analyses using a random-effects model. Covariates included: surgery type (upper abdominal surgery, upper abdominal surgery), TAPB approach (subcostal approach, lateral approach, and posterior approach), and local anesthetic type (bupivacaine, ropivacaine). Meta-regression analyses were performed by Stata 18.0 (Stata Statistical Software Release 18; StataCorp, College Station, TX, USA, 2023).

## Results

### Search results

**Figure 1:**
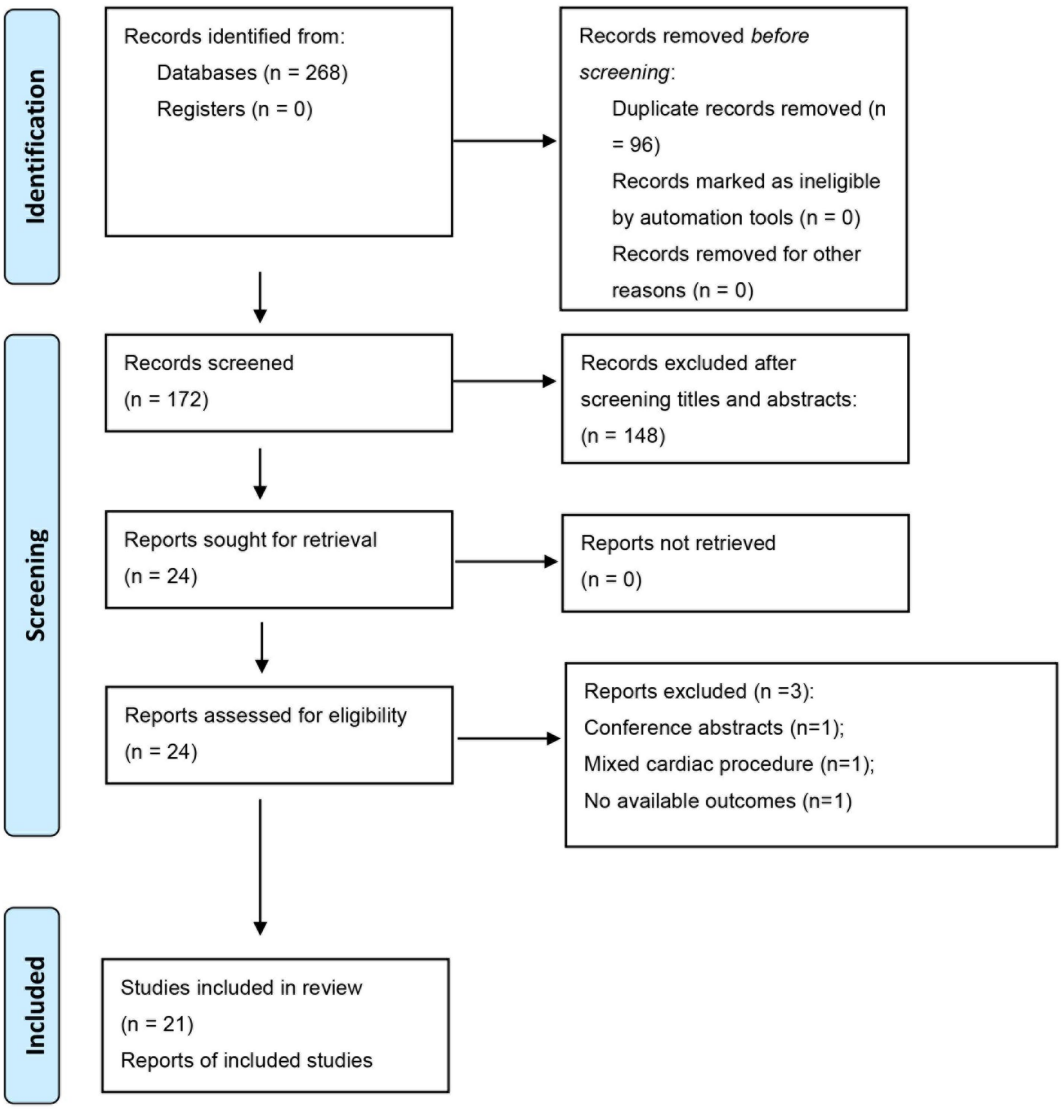
The inclusion process of the literature search.

The systematic retrieval across four biomedical databases (PubMed, Embase, Cochrane library, Web of science) yielded 268 candidate records as of October 31, 2024. First, we excluded 96 duplicate publications. Subsequent title/abstract screening eliminated 148 records due to: Non-target population (e.g., pediatric/emergency surgeries; *n* = 67), Intervention mismatch (combined regional techniques; *n* = 41), Study design ineligibility (non-RCTs; *n* = 40). Then, full-text appraisal of the remaining 24 articles applied the PICOS exclusion hierarchy: protocol violations (*n* = 1: mixed cardiac procedure) (16); insufficient outcome reporting (*n* = 1) (17); publication type exclusion (*n* = 1: conference abstract without peer review) (18). The final synthesis incorporated 21 RCTs spanning 2019–2024 (9–11, 19–36), with detailed selection dynamics visualized in the PRISMA 2020 flowchart (Figure 1).

### Risk of bias

**Figure 2:**
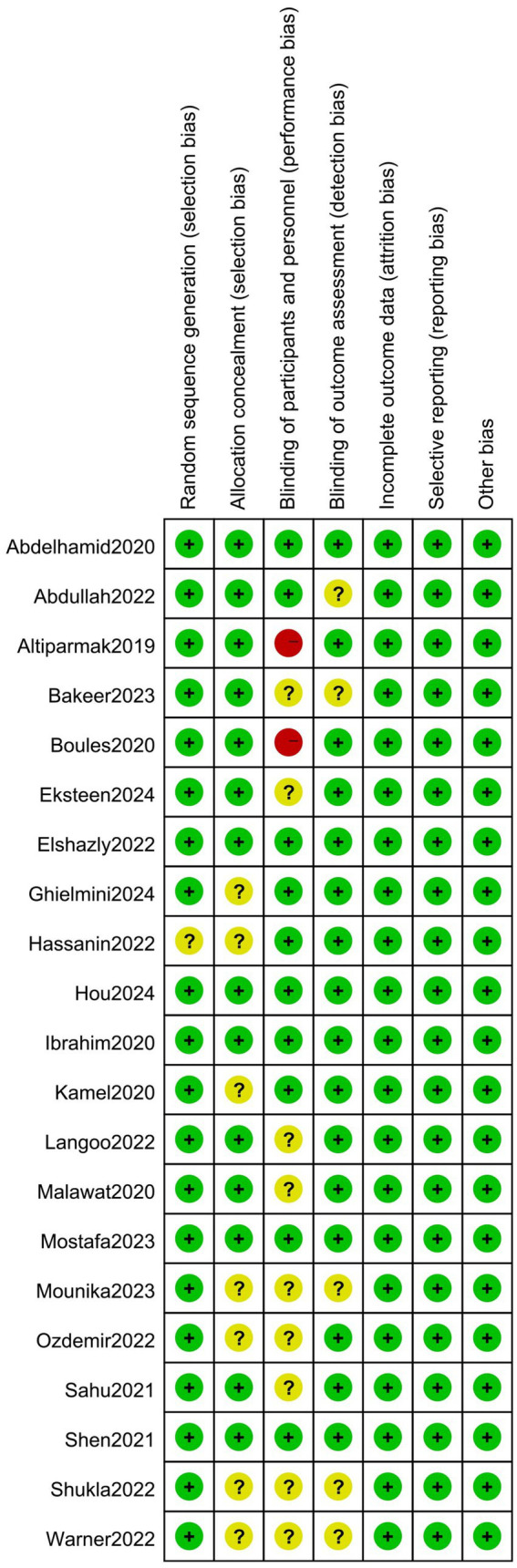
The risk bias assessment of all included studies.

All but one of the included studies explicitly reported the randomization methods employed (25). Seven studies (33.3%) inadequately documented concealment protocols, precluding assessment of selection bias mitigation. Eleven trials (52.4%) failed to implement double-blinding procedures, compromising participant-researcher blinding integrity. Five studies (23.8%) neglected to report outcome assessor blinding status, introducing potential measurement inaccuracies. Figure 2 presents a summary of the bias risk for the included studies.

### Outcomes

#### Primary outcome

##### Postoperative 2-h pain score

**Figure 3:**
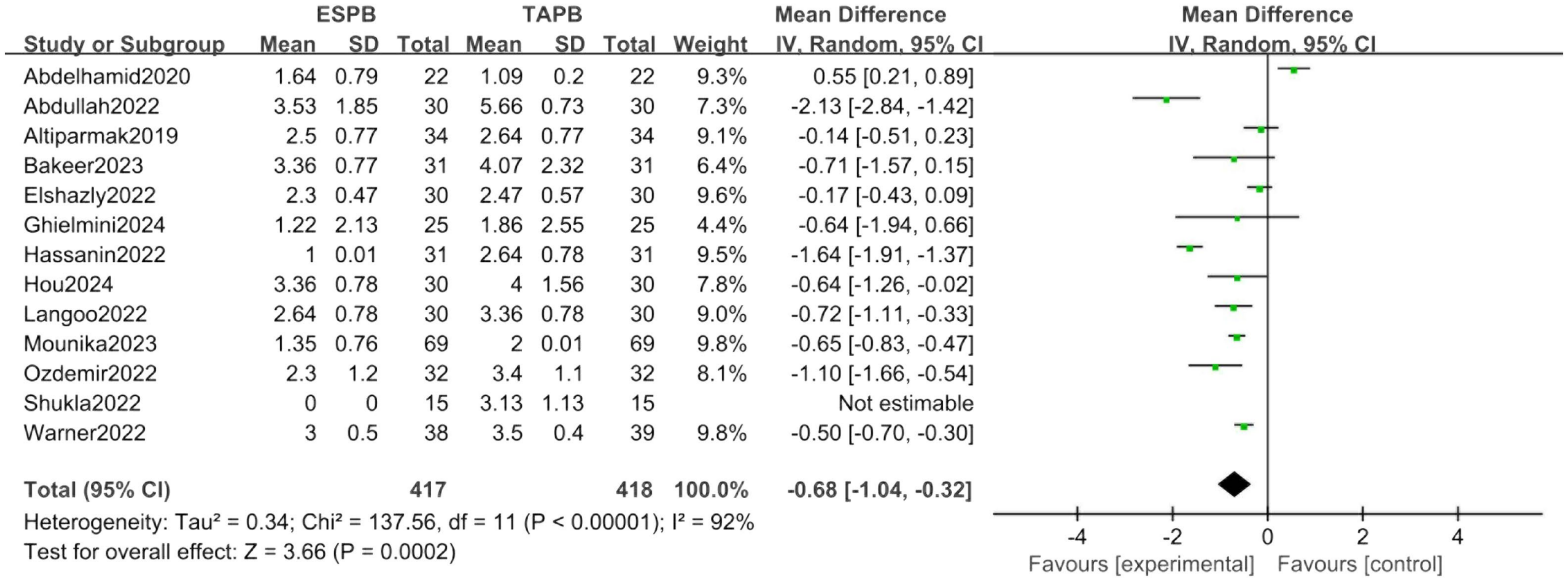
Forest plot of postoperative 2-h pain score between ESPB and TAPB groups. (ESPB, erector spinae plane block; TAPB, transversus abdominis plane block).

Thirteen trials reported postoperative 2-h pain score. The forest plot indicated a significant lower pain score in ESPB group (MD = −0.68, 95% CI [−1.04, −0.32], *p* < 0.05, *I*^2^ = 92%, Figure 3), highlighting substantial heterogeneity among the studies.

#### Secondary outcomes

##### Postoperative 4-h pain score

**Figure 4:**
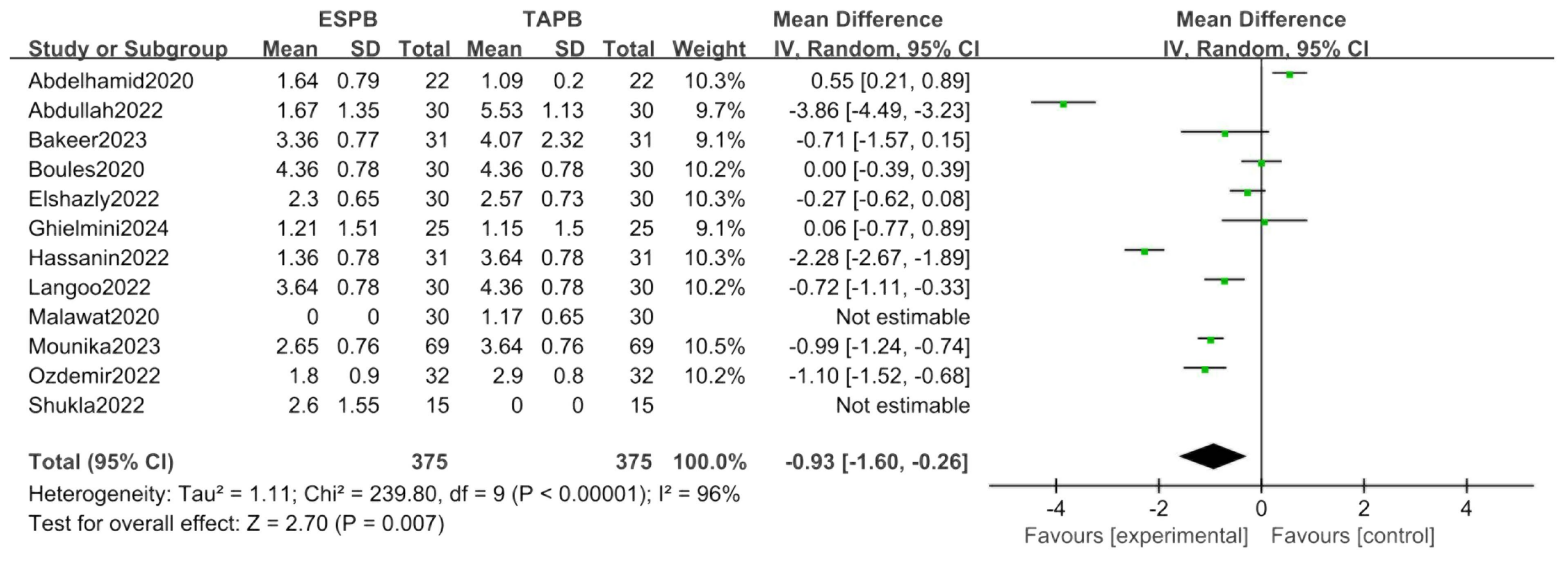
Forest plot of postoperative 4-h pain score between ESPB and TAPB groups. (ESPB, erector spinae plane block; TAPB, transversus abdominis plane block).

Data from 12 trials demonstrated a significant reduction in pain score for the ESPB group (MD = −0.93, 95% CI [−1.60, −0.26], *p* < 0.05), with substantial heterogeneity (*I*^2^ = 96%; Figure 4).

##### Postoperative 6-h pain score

**Figure 5:**
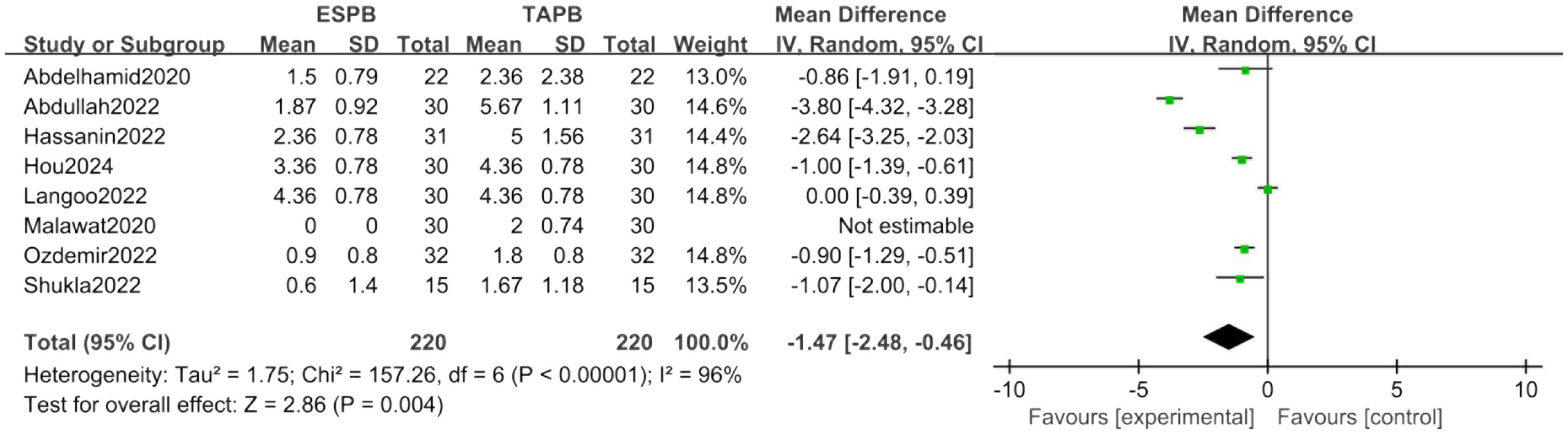
Forest plot of postoperative 6-h pain score between ESPB and TAPB groups. (ESPB, erector spinae plane block; TAPB, transversus abdominis plane block).

Eight trials revealed superior analgesic efficacy in the ESPB group (MD = −1.47, 95% CI [−2.48, −0.46], *p* < 0.05), accompanied by significant heterogeneity (*I*^2^ = 96%; Figure 5).

##### Postoperative 8-h pain score

**Figure 6:**
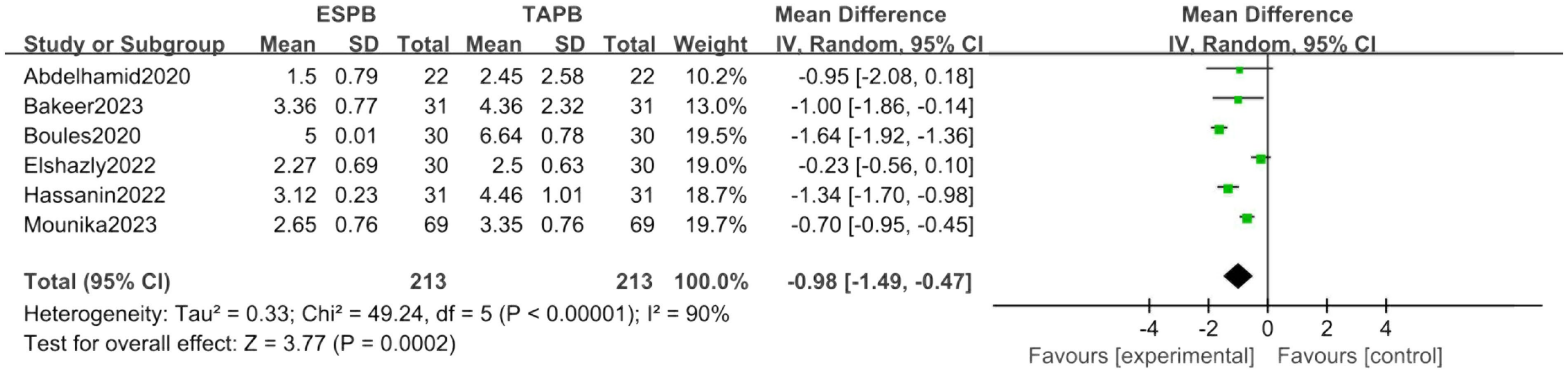
Forest plot of postoperative 8-h pain score between ESPB and TAPB groups. (ESPB, erector spinae plane block; TAPB, transversus abdominis plane block).

Analysis of six trials confirmed sustained analgesic superiority of ESPB (MD = -0.98, 95% CI [−1.49, −0.47], *p* < 0.05), despite marked heterogeneity (*I*^2^ = 90%; Figure 6).

##### Postoperative 12-h pain score

Fourteen studies indicated reduced pain scores in the ESPB cohort (MD = −0.73, 95% CI [−1.32, −0.13], *p* < 0.05), with pronounced heterogeneity (*I*^2^ = 97%; Supplementary Figure 1).

##### Postoperative 24-h pain score

Persistent analgesic benefits were observed in 14 trials for ESPB (MD = −0.51, 95% CI [−0.82, −0.20], *p* < 0.05), maintaining high heterogeneity (*I*^2^ = 93%; Supplementary Figure 2).

##### Postoperative opioid consumption

**Figure 7:**
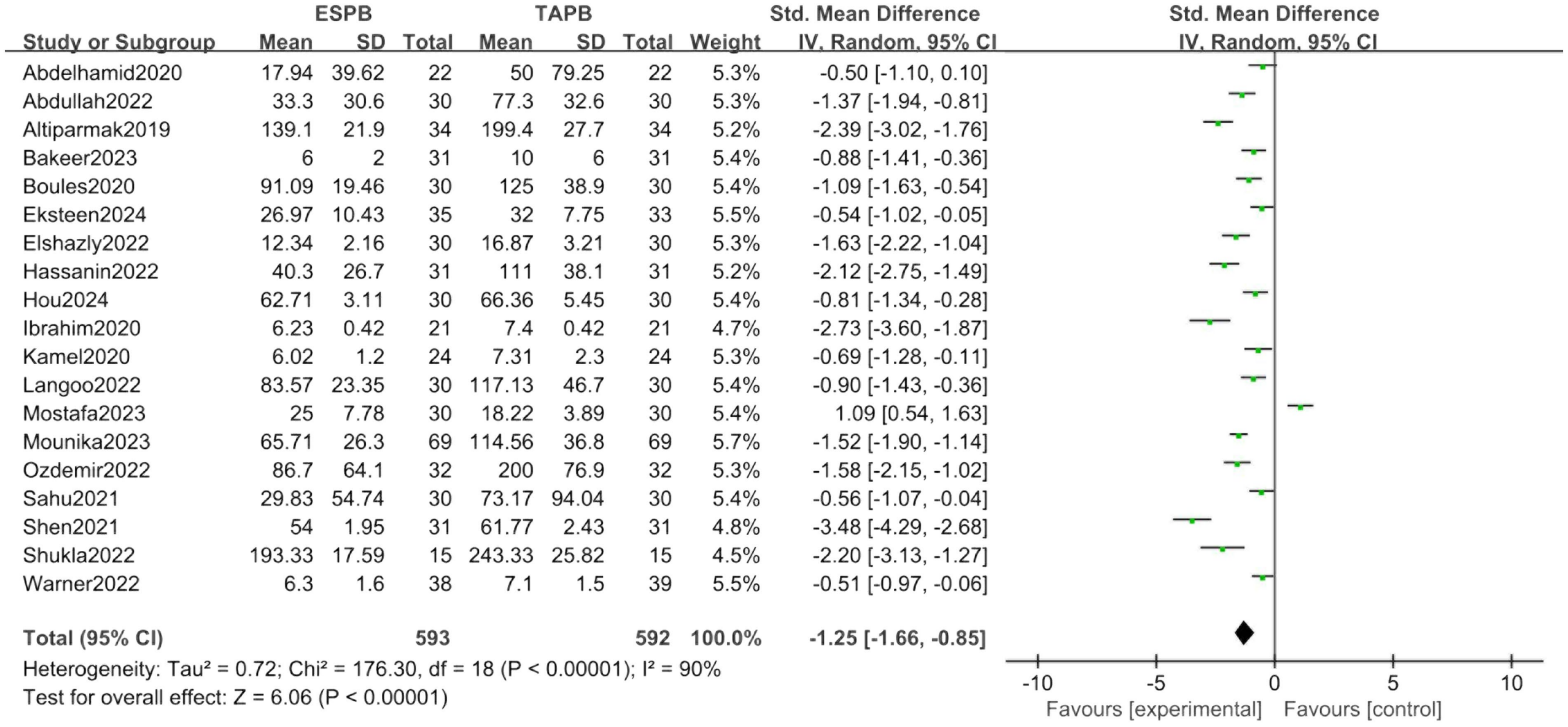
Forest plot of postoperative opioid consumption between ESPB and TAPB groups. (ESPB, erector spinae plane block; TAPB, transversus abdominis plane block).

Nineteen trials assessed postoperative opioid consumption. The forest plot revealed a significantly lower consumption in the ESPB group (SMD = −1.25, 95% CI [−1.66, −0.85], *p* < 0.05, *I*^2^ = 90%, Figure 7), indicating low heterogeneity among the studies.

##### Adverse events

**Figure 8:**
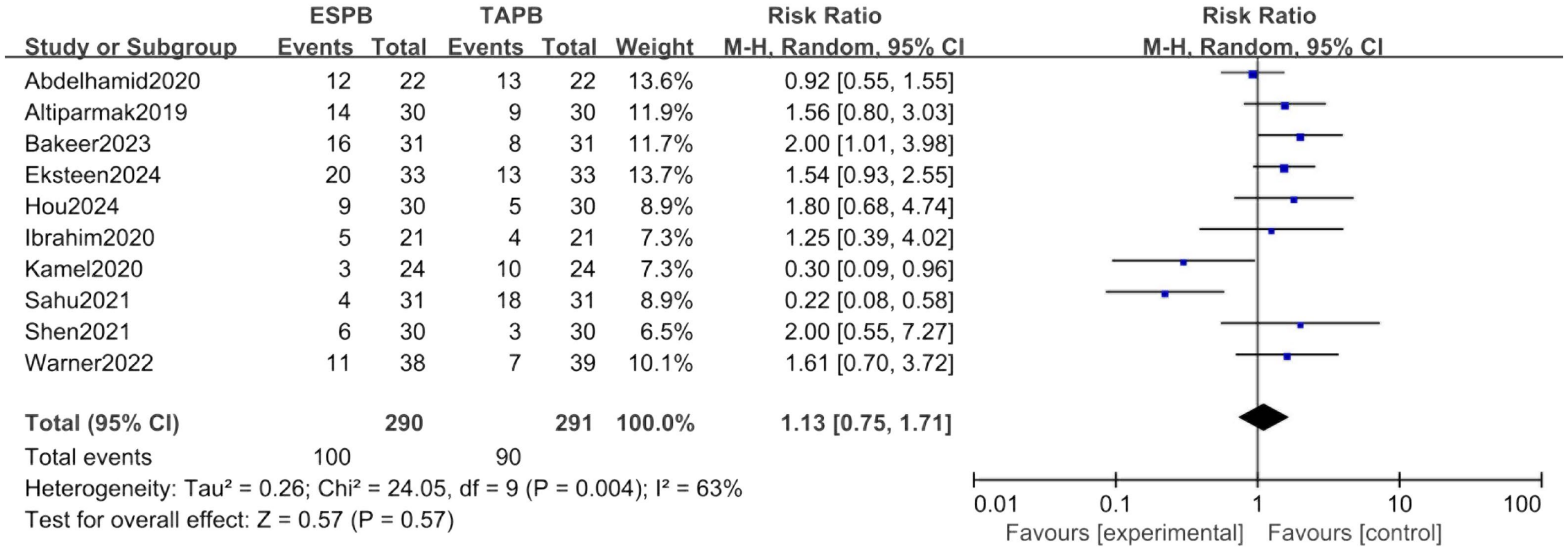
Forest plot of the incidence of PONV between ESPB and TAPB groups. (PONV, postoperative nausea and vomiting; ESPB, erector spinae plane block; TAPB, transversus abdominis plane block).

Ten trials examined the incidence of PONV. The forest plot demonstrated no significant incidence between two groups (RR = 1.13, 95% CI [0.75, 1.71], *p* > 0.05, *I*^2^ = 63%, Figure 8). No operative-related event was reported in both groups.

##### Meta-regression analysis

Meta-regression revealed no significant associations between the prespecified covariates (surgery type, TAPB approach, or local anesthetic) and heterogeneity in postoperative *2-h* pain scores (all *p*-values > 0.05) (Table 1).

**Table 1:** The details of included studies.

Study	Age	Sample size	ASA scale	Type of surgery	ESPB group	TAPB group	Anesthesia	PCIA
Abdelhamid et al. (19)	18–59	44	II-III	Laparoscopic sleeve gastrectomy	Location: 2–3 cm lateral to T9; Local anesthetic: 0.25% bupivacaine 15 mL on each side.	Location: oblique subcostal approach; Local anesthetic: 30 mL of 0.25% bupivacaine on each side.	General anesthesia	None
Abdullah et al. (20)	18–65	60	I-II	Ovarian cancer surgery	Location: 3 cm lateral to T10; Local anesthetic: 20 mL 0.25% bupivacaine on each side.	Location: at the level of the umbilicus; Local anesthetic: 20 mL 0.25% bupivacaine on each side	General anesthesia	Fentanyl
Altiparmak et al. (21)	18–70	68	I-II	Laparoscopic cholecystectomy	Location: 3 cm lateral to T7; Local anesthetic: 20 mL 0.375% bupivacaine on each side.	Location: oblique subcostal approach; Local anesthetic: 20 mL 0.375% bupivacaine on each side.	General anesthesia	Tramadol
Bakeer et al. (22)	18–65	62	II-III	Abdominal surgery	Not mentioned.	Not mentioned.	Not mentioned	Morphine
Boules et al. (23)	18–40	60	I-II	Cesarean	Location: 3 cm lateral to T10; Local anesthetic: 20 mL 0.5% bupivacaine on each side.	Location: between the costal margin and iliac crest Local anesthetic: 20 mL 0.5% bupivacaine on each side.	Spinal anesthesia	None
Eksteen et al. (11)	>18	66	I-III	Cesarean	Location: 2–3 cm lateral to T9; Local anesthetic: 20 mL 0.25% bupivacaine on each side.	Location: between the costal margin and iliac crest; Local anesthetic: 20 mL 0.25% bupivacaine on each side.	Spinal anesthesia	Morphine
Elshazly et al. (24)	18–60	60	II-III	Laparoscopic cholecystectomy or paraumbilical hernia repair	Location: 3 cm lateral to T5; Local anesthetic: 20 mL 0.25% bupivacaine on each side.	Location: oblique subcostal approach; Local anesthetic: 20 mL 0.25% bupivacaine on each side.	General anesthesia	None
Ghielmini et al. (10)	>18	50	I-II	Robot assisted hernia repair	Location: at the level of T10; Local anesthetic: 30 mL 0.2% ropivacaine on each side.	Location: in the triangle of Peti; Local anesthetic: 30 mL 0.2% ropivacaine on each side.	General anesthesia	None
Hassanin et al. (25)	20–50	62	I-III	Emergency laparotomy	Location: 3 cm lateral to T8; Local anesthetic: 20 mL 0.25% bupivacaine on each side.	Location: between the costal margin and iliac crest; Local anesthetic: 20 mL 0.25% bupivacaine on each side.	General anesthesia	None
Hou et al. (9)	18–65	60	I-III	Laparoscopic radical surgery	Location: 2–3 cm lateral to T9; Local anesthetic: 20 mL 0.2% ropivacaine on each side.	Location: oblique subcostal approach; Local anesthetic: 20 mL 0.2% ropivacaine on each side.	General anesthesia	Sufentani
Ibrahim et al. (26)	20–60	42	I-III	Laparoscopic cholecystectomy	Location: 3 cm lateral to L3; Local anesthetic: 20 mL 0.25% bupivacaine on each side.	Location: oblique subcostal approach; Local anesthetic: 20 mL 0.25% bupivacaine on each side.	General anesthesia	Tramadol
Kamel et al. (27)	40–60	48	I-II	Open total abdominal hysterectomy	Location: 3 cm lateral to T9; Local anesthetic: 20 mL 0.375% bupivacaine on each side.	Location: between the costal margin and iliac crest; Local anesthetic: 20 mL 0.375% bupivacaine on each side.	General anesthesia	None
Langoo et al. (28)	18–40	60	II	Cesarean	Location: 3 cm lateral to T10; Local anesthetic: 20 mL 0.2% ropivacaine on each side.	Location: between the costal margin and iliac crest; Local anesthetic: 20 mL 0.2% ropivacaine on each side.	Spinal anesthesia	Diclofenac
Malawat et al. (29)	18–80	60	I-III	Cesarean	Location: 3 cm lateral to T9; Local anesthetic: 0.2% ropivacaine 0.2 mL/kg on each side.	Location: between the costal margin and iliac crest; Local anesthetic: 0.2% ropivacaine 0.2 mL/kg on each side.	Spinal anesthesia	None
Mostafa et al. (30)	18	60	I-III	Open liver resection surgery	Location: 3 cm lateral to T7; Local anesthetic: 20 mL 0.25% bupivacaine on each side.	Location: oblique subcostal approach; Local anesthetic: 20 mL 0.25% bupivacaine on each side.	General anesthesia	None
Mounika et al. (31)	18–70	138	I-II	Laparoscopic cholecystectomy	Location: the level of T7; Local anesthetic: 20 mL 0.2% ropivacaine on each side.	Location: oblique subcostal approach; Local anesthetic: 20 mL 0.2% ropivacaine on each side.	General anesthesia	None
Ozdemir et al. (32)	18–64	64	I-III	Laparoscopic cholecystectomy	Location: 3 cm lateral to T7; Local anesthetic: 10 mL 0.25% bupivacaine and 10 mL of 2% prilocaine on each side.	Location: oblique subcostal approach; Local anesthetic: 10 mL 0.25% bupivacaine and 10 mL of 2% prilocaine on each side.	General anesthesia	None
Qi-Hong et al. (33)	65	62	I-III	Laparoscopic colorectal surgery	Location: 2–3 cm lateral to T9; Local anesthetic: 20 mL 0.25% ropivacaine on each side.	Location: oblique subcostal approach; Local anesthetic: 20 mL 0.25% ropivacaine on each side.	General anesthesia	Sufentanil
Sahu et al. (34)	18–70	60	I-II	Laparoscopic cholecystectomy	Location: 2–3 cm lateral to T7; Local anesthetic: 20 mL 0.2% ropivacaine on each side.	Location: oblique subcostal approach; Local anesthetic: 20 mL 0.2% ropivacaine on each side.	General anesthesia	None
Shukla et al. (35)	35–60	30	I-II	Open total abdominal hysterectomy	Location: 3 cm lateral to T9; Local anesthetic: 20 mL 0.25% bupivacaine on each side.	Location: between the costal margin and iliac crest; Local anesthetic: 20 mL 0.25% bupivacaine on each side.	General anesthesia	None
Warner et al. (36)	18	77	I-IV	Laparoscopic hysterectomy	0.125% bupivacaine 20 mL at T8 and 20 mL at T12 on each side.	0.125% bupivacaine 20 mL for the subcostal TAP and 20 mL for the posterior TAP on each side.	General anesthesia	None

ASA, American society of anesthesiologists; ESPB, Erector spinae plane block; TAPB, Transversus abdominis plane block; PCIA, Patient controlled intravenous analgesia.

##### GRADE result

Table 2 shows the summary of the GRADE assessment.

**Table 2:** The summary of GRADE for included studies.

Outcome	Included studies (*n*)	Patients (*n*)	Quality of evidence	Reasons
Postoperative 2-h pain score	13	835	⨁⨁⨁◯ MODERATE	“Inconsistency” was downgraded to “serious.”
Postoperative 4-h pain score	12	750	⨁⨁⨁◯ MODERATE	“Inconsistency” was downgraded to “serious.”
Postoperative 6-h pain score	8	440	⨁⨁⨁◯ MODERATE	“Inconsistency” was downgraded to “serious.”
Postoperative 8-h pain score	6	426	⨁⨁⨁◯ MODERATE	“Inconsistency” was downgraded to “serious.”
Postoperative 12-h pain score	14	878	⨁⨁⨁◯ MODERATE	“Inconsistency” was downgraded to “serious.”
Postoperative 24-h pain score	14	878	⨁⨁⨁◯ MODERATE	“Inconsistency” was downgraded to “serious.”
Postoperative opioid consumption	19	1,185	⨁⨁◯◯ LOW	“Imprecision” and “Inconsistency” were downgraded to “serious.”
Incidence of PONV	10	581	⨁⨁⨁◯ MODERATE	“Inconsistency” was downgraded to “serious.”

PONV, postoperative nausea and vomiting.

## Discussion

Our meta-analysis investigated the safety and effectiveness of ESPB in abdominal surgeries while comparing with TAPB. The results showed that ESPB significantly decreased postoperative pain scores and opioid consumption.

Abdominal surgical pain originates from multiple sources: incisional discomfort, visceral nociception, tissue trauma, CO₂ insufflation-induced shoulder pain, and phrenic nerve irritation (37). This multimodal pathophysiology results in concurrent somatic and visceral pain perception. Effective multimodal analgesia enhances patient satisfaction, accelerates functional recovery, reduces hospitalization duration, and decreases thromboembolic risks through improved early mobilization (38). Existing evidence confirms the analgesic efficacy of both TAPB and ESPB in abdominal surgical settings. However, contemporary meta-analyses present discordant conclusions regarding their comparative effectiveness. Thus, we conducted this systematic review with meta-analysis incorporating 21 randomized controlled trials (*N* = 1,293 patients) to compare the efficacy of ESPB versus TAPB for postoperative analgesia.

Our meta-analysis demonstrated the analgesic superiority of ESPB over TAPB, evidenced by significantly reduced postoperative pain scores and lower opioid consumption. While the precise mechanism of ESPB’s analgesic action remains debated within the scientific community, emerging cadaveric and radiological evidence suggests dual neural targeting - simultaneously engaging both ventral and dorsal rami of spinal nerves through fascial compartment diffusion (39, 40). This bidirectional blockade achieves comprehensive somatic-visceral pain control, a mechanistic advantage over TAPB’s limited anterior ramus inhibition.

We further evaluated the safety of ultrasound-guided ESPB, and none of the included studies reported procedure-related complications. Current literature suggests that severe complications occur in fewer than 0.02% (2 per 10,000) of cases (39), with documented adverse events involving motor nerve blockade, lung puncture (pneumothorax), accidental vascular puncture, and systemic toxic reactions. The procedure’s safety advantage stems from its anatomical approach, where injectates are deliberately positioned distal to vulnerable neurovascular structures like the spinal canal, pleural membranes, and major blood vessels.

Our study has limitations to consider. First, the significant differences in pain scores and opioid use across postoperative time points may stem from varied surgical and pain management approaches. Although we accounted for this using statistical methods, results should be interpreted carefully. Second, a small number of included studies exhibited a high risk of bias.

## Conclusion

This systematic review and meta-analysis demonstrates that ultrasound-guided ESPB provides superior postoperative analgesia compared to TAPB. Our meta-regression did not identify surgery type, TAPB approach, or local anesthetic properties as sources of heterogeneity, future research should prioritize prospective studies with stratified designs to evaluate these covariates in homogenous surgical populations.

## Data Availability

The original contributions presented in the study are included in the article/Supplementary material, further inquiries can be directed to the corresponding author.
