# Supplementary material for: Comparison of the efficiency of ultrasound-guided ESPB and TAPB on postoperative analgesia: a system review and meta-analysis
Source: Front Med (Lausanne). 2025 May 26;12:1595778. doi: 10.3389/fmed.2025.1595778 (PMC12146374; doi:10.3389/fmed.2025.1595778)
Supplement: Supplementary file 3 [file Table_1.DOCX]

Search strategy for Pubmed

("abdominal muscles"[MeSH Terms] OR ("abdominal"[All Fields] AND "muscles"[All Fields]) OR "abdominal muscles"[All Fields] OR ("transversus"[All Fields] AND "abdominis"[All Fields]) OR "transversus abdominis"[All Fields]) AND ("aircraft"[MeSH Terms] OR "aircraft"[All Fields] OR "plane"[All Fields] OR "planes"[All Fields]) AND ("block"[All Fields] OR "blocked"[All Fields] OR "blocking"[All Fields] OR "blockings"[All Fields] OR "blocks"[All Fields]) AND (("erector"[All Fields] OR "erectores"[All Fields] OR "erectors"[All Fields]) AND "spinae"[All Fields] AND ("aircraft"[MeSH Terms] OR "aircraft"[All Fields] OR "plane"[All Fields] OR "planes"[All Fields]) AND ("block"[All Fields] OR "blocked"[All Fields] OR "blocking"[All Fields] OR "blockings"[All Fields] OR "blocks"[All Fields]))
